# Effects of extreme temperatures and recovery potential of *Gongolaria barbata* from a coastal lagoon in the northern Adriatic Sea: an *ex situ* approach

**DOI:** 10.1093/aob/mcae038

**Published:** 2024-03-14

**Authors:** Andrea Bilajac, Edi Gljušćić, Shannen Smith, Mirjana Najdek, Ljiljana Iveša

**Affiliations:** Ruđer Bošković Institute, Center for Marine Research, G. Paliaga 5, 52210 Rovinj, Croatia; Ruđer Bošković Institute, Center for Marine Research, G. Paliaga 5, 52210 Rovinj, Croatia; Ruđer Bošković Institute, Center for Marine Research, G. Paliaga 5, 52210 Rovinj, Croatia; Ruđer Bošković Institute, Center for Marine Research, G. Paliaga 5, 52210 Rovinj, Croatia; Ruđer Bošković Institute, Center for Marine Research, G. Paliaga 5, 52210 Rovinj, Croatia

**Keywords:** *Gongolaria*, *Cystoseira*, Adriatic Sea, lagoon, climate change, marine heatwaves, thermotolerance, morphology, physiology, recovery

## Abstract

**Background and Aims:**

Globally, rising seawater temperatures contribute to the regression of marine macroalgal forests. Along the Istrian coastline (northern Adriatic), an isolated population of *Gongolaria barbata* persists in a coastal lagoon, representing one of the last marine macroalgal forests in the region. Our objective was to examine the impact of extreme temperatures on the morphology and physiology of *G. barbata* and test its potential for recovery after simulating marine heatwave (MHW) conditions.

**Methods:**

We explored the occurrence of marine heatwaves in southern Istria, adjacent to the study area, in addition to extreme temperatures inside the area itself. Subsequently, we performed a thermotolerance experiment, consisting of a stress and recovery phase, in which we exposed *G. barbata* thalli to four extreme (28, 30, 32 and 34 °C) and one favourable (18 °C) temperature. We monitored morphological and physiological responses.

**Key Results:**

Our findings indicate a significant rise in frequency, duration and intensity of MHWs over decades on the southern Istrian coast. Experimental results show that *G. barbata* demonstrates potential for both morphological and physiological recovery after exposure to temperatures as high as 32 °C. However, exposure to 34 °C led to thallus decay, with limited ability to regenerate.

**Conclusions:**

Our results show that *G. barbata* has a remarkable resilience to long-term exposure to extreme temperatures ≤32 °C and suggest that short-term exposure to temperatures beyond this, as currently recorded inside the lagoon, do not notably affect the physiology or morphology of local *G. barbata*. With more MHWs expected in the future, such an adapted population might represent an important donor suitable for future restoration activities along the Istrian coast. These results emphasize the resilience of this unique population, but also warn of the vulnerability of marine macroalgal forests to rising seawater temperatures in rapidly changing climatic conditions.

## INTRODUCTION

Along with gradual warming trends across the global coastal ocean (Lima and Wethey, 2012; Johnson and Lyman, 2020), the frequency and intensity of marine heatwaves (MHWs) are on an increasing trajectory (Frölicher *et al.*, 2018; Oliver, 2019; Oliver *et al.*, 2021) as a consequence of anthropogenic climate change. Thermal tolerance thresholds of organisms are being exceeded more frequently and by greater magnitudes (Frölicher *et al.*, 2018; Oliver *et al.*, 2018; Sen Gupta *et al.*, 2020), with MHWs exhibiting various impacts on species reproduction and range shifts, in addition to the establishment of non-native species (Smith *et al.*, 2023). Moreover, the increasing frequency of MHWs has been linked to the rapid increase in mass mortality events worldwide (Hanley *et al.*, 2023), with the Mediterranean Sea ecosystem in particular being negatively impacted over the last two decades (Garrabou *et al.*, 2022; Estaque *et al.*, 2023). The most dramatic events in terms of geographical extent and number of affected species occurred in 1999 and 2003 along the northwestern Mediterranean Sea (Cerrano *et al.*, 2000; Perez *et al.*, 2000; Garrabou *et al.*, 2009). These two events affected >40 species from various taxa across thousands of kilometres of coastline. Additionally, during the 2015–2019 period the basin experienced mass mortality events that affected thousands of kilometres of coastline from the surface to 45 m of depth (affecting 50 taxa across eight phyla; Garrabou *et al.*, 2022).

Climate extremes and changes to the global water cycle are of crucial importance for the northern Adriatic Sea, i.e. the northernmost, shallowest and most productive part of the Mediterranean region (Bianchi and Morri, 2000). Temperatures currently decrease below 11 °C in winter (Bianchi, 2007) and approach 30 °C in summer. Owing to its complex circulation (Vilibić *et al.*, 2023), the northern Adriatic experiences periods of eutrophication (Degobbis *et al.*, 2000; Djakovac *et al.*, 2012), mucilage formation events (Giani *et al.*, 2005), hypoxia/anoxia events (Djakovac *et al.*, 2015) and harmful algal blooms (Facca *et al.*, 2014). Together with these stressors, invasions of allochthonous species (Shiganova and Malej, 2009; Iveša *et al.*, 2015) and sea urchin outbreaks (Iveša *et al.*, 2016) represent disturbances that potentially threaten biodiversity, including ecologically important macroalgal forests (Iveša *et al.*, 2016, 2021, 2022; Gljušćić *et al.*, 2023). The Mediterranean Sea is generally considered a relevant model to assess the ecological effects of climate change on marine biodiversity and to test potential adaptation and mitigation strategies that might be scaled up (Cramer *et al.*, 2018). Likewise, the importance and peculiarities of the northern Adriatic specifically make it a natural laboratory for studying endangered populations, their reintroduction and best conservation practices (Orlando-Bonaca *et al.*, 2021, 2022*a*; Gljušćić *et al.*, 2023; Lokovšek *et al.*, 2023).

Brown algal species of the genus *Cystoseira s.l.* play an important role as habitat builders (Gianni *et al.*, 2013; Blanfuné *et al.*, 2019) and ecosystem services providers, because their three-dimensional structure provides habitat and shelter for smaller algae, invertebrates and fish on rocky bottoms (Orlando-Bonaca and Lipej, 2005; Ballesteros *et al.*, 2009; Cheminée *et al.*, 2013; Pitacco *et al.*, 2014; Orlando-Bonaca *et al.*, 2022*b*). In recent decades, the decline of fucalean-dominated assemblages has been observed in different regions of the Mediterranean Sea as a result of habitat destruction (urbanization and extreme storm events), pollution, overgrazing (sea urchins and herbivorous fish) and global warming (Guidetti *et al.*, 2004; Sala *et al.*, 2012; Gianni *et al.*, 2013; Ling *et al.*, 2015; Mineur *et al.*, 2015; Bianchi *et al.*, 2017; Darmaraki *et al.*, 2019; Fabbrizzi *et al.*, 2020; Verdura *et al.*, 2021).

In our study, we focused on the thermal tolerance and resilience of the canopy-forming fucalean macroalga *Gongolaria barbata*, which dwells on the rocky bottom of the northern Adriatic (Ercegović, 1952; Iveša *et al.*, 2016, 2022). It belongs to the warm-temperate Mediterranean–Atlantic group of algae and is geographically widespread (Orfanidis, 1991; Falace *et al.*, 2005). Along the coastline studied here (Istrian coast, Croatia), historical data show that the occurrence of *G. barbata* and of other *Cystoseira s.l.* species fluctuates, with periods of high and lower cover, possibly related to eutrophication patterns and sea urchin occurrence and abundance (Iveša *et al.*, 2016). Since 2016, field observations have led us to hypothesize that *G. barbata* is once again in a regression phase, where its distribution is limited to few shallow sites and rockpools. One of the biggest and, possibly, the last well-developed forest is found in a shallow coastal lagoon, Šćuza (Pomer Bay), on the southern Istrian coast (Iveša *et al.*, 2022).

Biological responses to MHWs happen at the individual, population and community levels and intensify towards the warm trailing range edges of species distributions, while range-centre and cold-water range-edge populations remain largely unaffected (Smale *et al.*, 2019; Hanley *et al.*, 2023; Smith *et al.*, 2023). In recent years, there has been particular interest in researching marginal/extreme populations, with the aim of understanding how populations in more favourable habitats will respond to future climate scenarios (Kolzenburg, 2022), because they already experience extreme conditions expected in the future. Exploring those populations leads to potential knowledge of the physiological tolerance limits and environmental drivers of organismal abundance, species distributions and evolutionary processes (Brown *et al.*, 1995; Bridle and Vines, 2007). Shallow populations live close to their upper thermotolerance limits (Verdura *et al.*, 2021; Gómez-Gras *et al.*, 2022) and are thus greatly threatened by MHWs (Garrabou *et al.*, 2022).

Here, we simulated marine heatwave events of varying intensity for the purpose of defining the impact of extreme temperatures on *G. barbata* individuals, in addition to the species resilience and potential for recovery. We monitored temperatures in Šćuza Lagoon and, based on summer extremes, conducted an *ex situ* thermotolerance experiment, consisting of a stress phase and a recovery phase. We explored the long-term frequency, duration and intensity of MHWs on the southern Istrian coast to put the lagoonal conditions in a broader environmental context and establish realistic test conditions for investigating thermal performance. This experiment was conducted to explore: (1) whether temperature extremes measured in the Šćuza Lagoon have adverse effects on the morphological and physiological characteristics of *G. barbata*; and (2) whether the thalli of *G. barbata* are capable of regeneration after being reintroduced to favourable thermal conditions. This information will contribute to the general knowledge of the ecology of a canopy-forming species that thrives in a challenging thermal environment, providing valuable insight into future conservation and restoration actions of endangered canopy-forming species in the Adriatic Sea.

## MATERIALS AND METHODS

### Target species

*Gongolaria barbata* is considered to be eurythermal, typically tolerating temperatures ranging from 8 to 23 °C, with optimal development occurring between 8 and 16–17 °C (Ercegović, 1952), but appears to survive and tolerate temperatures well beyond that limit, from below freezing to >30 °C, at least in the short term (Orfanidis, 1991; Iveša *et al.*, 2022). Typically, *G. barbata* possesses a singular cauloid (but see Iveša *et al.*, 2022) capable of attaining a maximum length of 50 cm, measuring 3–5 mm in diameter, and can be identified by a discoidal holdfast and by a smooth and highly prominent apex. In conjunction with the primary and higher-order branches, certain individuals can extend up to a length of 1 m. *Gongolaria barbata* shows high morphological variability, cyclically dependent on season and geographically dependent on wave exposure and other environmental factors, such as light and temperature (Ercegović, 1952; Falace and Bressan, 2006; Sadogurska, 2021). Specifically, the size of receptacles and the shape and size of aerocysts show the greatest plasticity (Sadogurska, 2021). Fertility of the species has been observed primarily during the period from March to June, during spring and early summer (Cebrian *et al.*, 2021).

### Sampling site

**Fig. 1.**
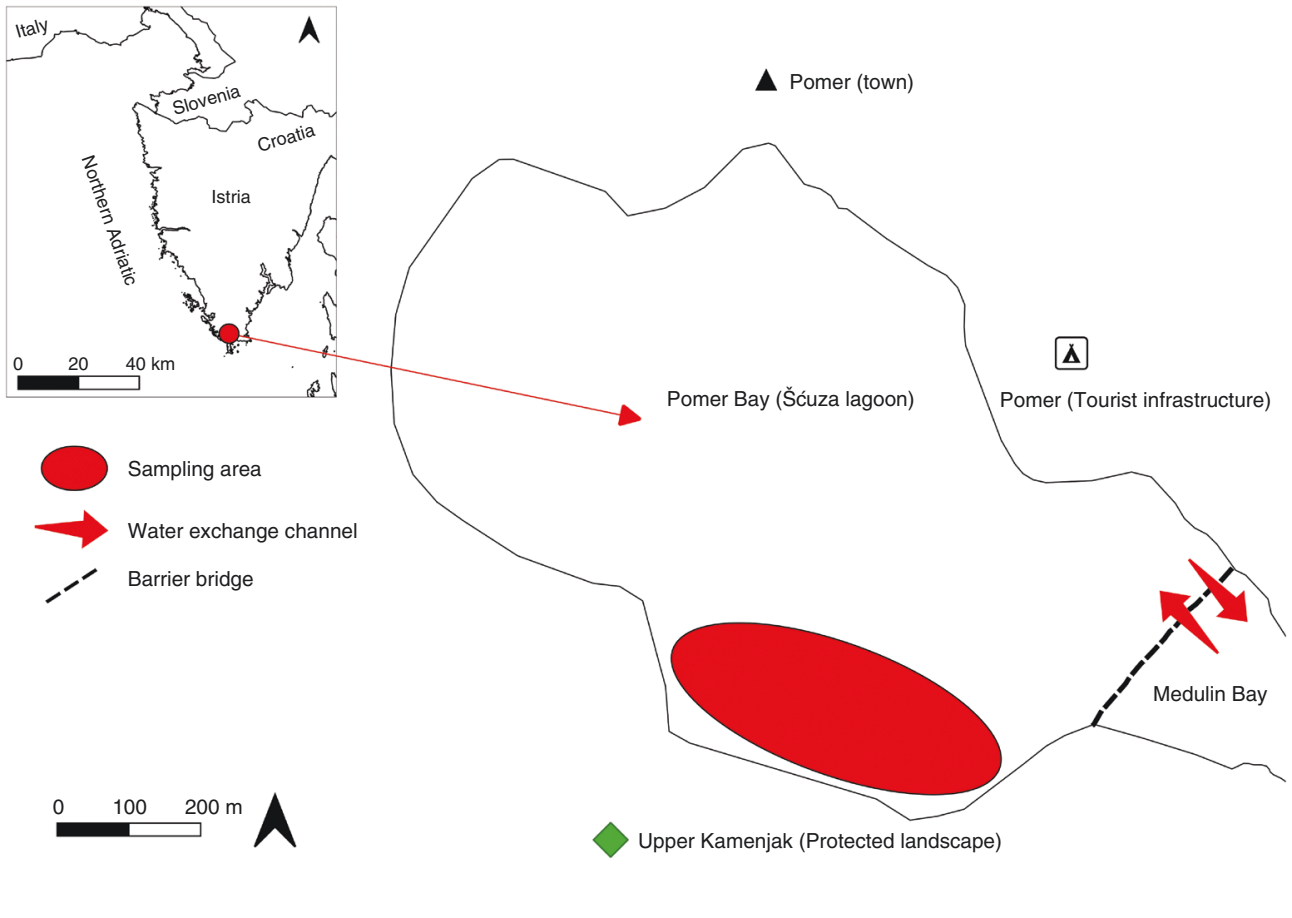
Map of the south Istrian coast showing the Šćuza Lagoon, the sampling site (red ellipse), the barrier bridge (dashed line) and water-exchange channels (thick red arrows).

Šćuza Lagoon, also known as Pomer Bay (Fig. 1), is located in southern Istria, in the northern Adriatic Sea (44°49ʹ13.3572″N, 13°53ʹ22.7400″E; European Environment Agency, 2021). It is a 68.6 ha shallow coastal lagoon, ≤1.5 m deep, with rocky, muddy and sandy bottom types inhabited by a euryhaline and eurythermal community. The dominant habitat-forming species are the seagrass *Cymodocea nodosa* and fucalean alga *G. barbata* (Iveša *et al.*, 2022). A similar coastal lagoon (Stjuža), both in name and in ecological characteristics, is located in Strunjan, in neighbouring Slovenia, and also represents the same habitat type but with a different dominant canopy-forming species, namely *Cystoseira foeniculacea* (Battelli and Catra, 2021, 2023).

Seasonal temperature trends in the lagoon generally follow those of the northern Adriatic; however, in both the upper and lower thermal range there are extreme fluctuations that are not recorded outside of the lagoon. In the lagoon, temperatures fluctuate from <0 °C in winter to >34 °C in summer (Iveša *et al.*, 2022), rendering it a unique thermal anomaly along the Istrian coast (Supplementary Data Fig. S1) and a useful natural laboratory for investigating the effects of extreme temperatures on *G. barbata*. Such natural laboratories are already recognized as analogues for future ocean conditions in coral research (Camp *et al.*, 2019; Maggioni *et al.*, 2021), in addition to being resilience hotspots for naturally stress-resistant coral species/communities (Riegl *et al.*, 2012; Palumbi *et al.*, 2014) and refugia from climate change stressors (Kavousi and Keppel, 2018; Kapsenberg and Cyronak, 2019; Mies *et al.*, 2020).

### Temperature conditions within and beyond the lagoon

The temperature at the sampling site was monitored continuously using a HOBO Pendant^®^ Temperature/Light 64K Data Logger, positioned 0.5 m below the sea surface, from 1 January 2020 to 31 December 2022, logging every 30 min. To analyse the data, daily maximum temperature values were extracted from the entire dataset. These values were then subjected to the ‘exceedance’ function, available in the heatwaveR package (Schlegel and Smit, 2018). In the function, thresholds of 28, 30, 32 and 34 °C were specified to identify instances when the temperature surpassed these limits. In the ‘exceedance’ function, a minimum duration of 5 days consecutively was set for the thresholds of 28, 30 and 32 °C, and a minimum duration of 1 day was considered for the 34 °C threshold. This approach allowed for the visual identification of periods when temperature remained above a specific threshold for the given duration. Furthermore, the data were grouped by year and temperature, and a summary was generated indicating the total number of days exceeding each threshold for each year. This summary provided a later visualization of the duration of specified events, offering insights into the frequency and intensity of temperature fluctuations over the years. Furthermore, the warmest periods recorded in Šćuza in the year 2022 were visualized using boxplots to explain the daily temperature range and fluctuations properly (Supplementary Data Fig. S2).

For the purpose of describing temperature conditions outside the lagoon, satellite-derived sea surface temperature (SST) data (OISST v.2) were downloaded from the ERDDAP data server (Simons and Chris, 2023). Pixels from the southern Istrian coast (latitude from 44°22ʹ30″ to 44°52ʹ30″N and longitude from 13°30ʹ0″ to 14°7ʹ30″E) were averaged per time point (daily average values) between 1983 and the end of 2022. *In situ* and SST daily mean temperatures were overlaid in the period from 2020 to the end of 2022, in order to visualize the different conditions inside and outside the lagoon. Moreover, to explore the long-term trend (intensity and duration) of MHWs outside the lagoon, SST data were subjected to the ‘ts2clm’, ‘detect event’ and ‘lolliplot’ functions from the heatwaveR package (Schlegel and Smit, 2018). The ‘ts2clm’ function allowed for determining the seasonal climatology and the 90th percentile threshold for calculating MHWs according to the definition by Hobday *et al.* (2016, 2018). The ‘detect_event’ function allowed for the detection of the duration and intensity of MHWs, and the ‘lolli_plot’ function allowed for visualization of the duration and intensity of MHWs in the period from the beginning of 1983 to the end of 2022.

### Experimental set-up

**Fig. 2.**
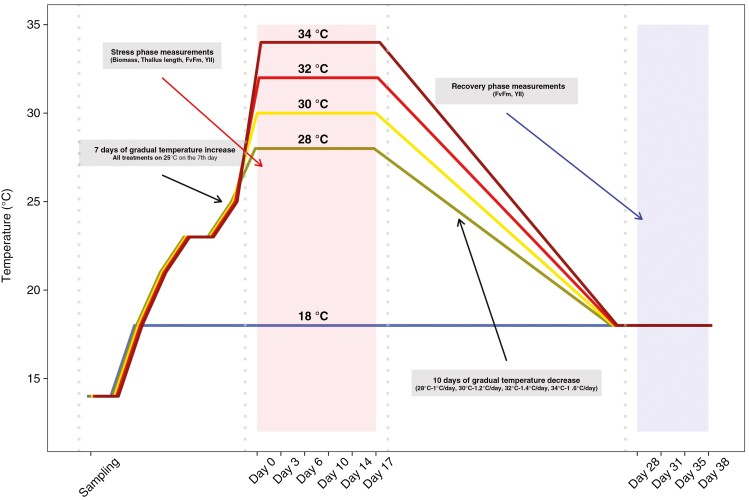
Schematic visualization of treatments: control conditions at 18 °C and extreme conditions at 28, 30, 32 and 34 °C. During first 7 days after sampling, the temperature was increased to 25 °C, and on day 8 it was increased in different treatments to designated temperature conditions. The stress phase was followed by 10 days of gradual temperature decrease and by the recovery phase.

On 7 March 2023, 45 individuals were collected randomly from the shallow coastal lagoon Šćuza (Fig. 1) and placed carefully in stone basins containing natural seawater (14 °C), where they were held until 9 March 2023. The individuals were fastened onto limestone tiles (10 cm × 10 cm × 2 cm) using elastic bands and later allocated to 24 L glass tanks containing filtered seawater and held at a constant temperature of 18 °C. Four different MHW scenarios were then simulated. A control temperature of 18 °C was chosen because this is when the species is at its vegetation peak within the lagoon, and maximum temperatures (28, 30, 32 and 34 °C) were chosen based on observed summer conditions during which the species is aestivating. The experimental set-up consisted of three separate tanks for each treatment, with three individuals housed within each tank (Supplementary Data Fig. S3). Over the following days, the temperature was maintained at 18 °C in the control tanks, while in other treatments it was increased up to 21 °C on 10 March 2023, up to 23 °C on 11 March 2023 and up to 25 °C on 13 March 2023. On 14 March 2023, the temperature of the four extreme treatments was increased up to 28, 30, 32 or 34 °C (Fig. 2). Then, initial measurements of biomass, morphometry and physiology were performed.

The experiment lasted for 38 days, including both the stress and recovery phases. The stress phase lasted for 17 days, during which the specimens were exposed to designated stress conditions. The recovery phase was characterized by a gradual reduction in temperature to control conditions, beginning on day 18 and lasting for 10 days (1 °C day^−1^, 28 °C treatment; 1.2 °C day^−1^, 30 °C treatment; 1.4 °C day^−1^, 32 °C treatment; and 1.6 °C day^−1^, 34 °C treatment; Fig. 2), after which the specimens were held at 18 °C for the remaining 10 days.

Biomass and thallus length measurements were conducted exclusively during the stress phase (days 0, 3, 6, 10, 14 and 17), whereas effective quantum yield (*YII*) and maximum quantum yield (*Fv*/*Fm*) measurements were obtained throughout both the stress and recovery phases (days 0, 3, 6, 10, 14, 17, 28, 31, 35 and 38; Fig. 2). At each measurement time point, samples were removed carefully from tanks and dried gently using a paper towel to remove excess water. Individuals were then photographed using high-resolution digital cameras to record any structural damage. Wet biomass and thallus length were then measured, followed by the assessment of *YII*. In the case of *Fv*/*Fm*, the specimens were subjected to a dark adaptation period of 15 min, after which a single measurement per individual was taken in dark conditions. Both quantum yield measurements were performed using MINI-PAM-II (Heinz Walz GmbH), measuring the fluorescence on the branches. Biomass and thallus length were assessed as percentages by comparing the measurements of each individual at a specific time point with their respective initial values. These changes are depicted such that the day 0 measurement is set as 100 % for both biomass and thallus length, while subsequent time points indicate the percentage of biomass and thallus length that remained relative to the initial measurements.

The temperature of 18 °C was maintained using the seawater temperature controllers (Teco TK500) and an air-conditioning unit. Higher temperature conditions were regulated with 100 W aquarium heaters. To maintain water quality, 50 % of the seawater in each tank was changed weekly. The natural seawater designated for volume replacement was heated to 25 °C and stored in a separate tank. Fluorescent tubes (Phillips Master TL-D 36 W/865, 6000 K) were used as light sources, providing an approximate irradiance of 50 μmol photons m^−2^ s^−1^, and the photoperiod followed a cycle of 12 h of light and 12 h of darkness.

### Statistical analysis

A linear mixed-effects model was used to investigate the effect of temperature on biomass, and a generalized linear mixed-effects model with a Poisson error distribution and a logit link function was used to analyse the effects on thallus length, *Fv*/*Fm* and *YII*. Temperature was fitted as a fixed factor, and time was incorporated as a cross-random factor. Additionally, a second random term, individuals nested within tanks, was included.

To explore the effect of the fixed factor, a type II Wald χ^2^ test was applied to each fitted model. Furthermore, Tukey’s post-hoc tests were conducted to determine specific differences between temperature treatments. All analyses were performed using the statistical environment R (R Core Team, 2023), with models fitted using the lme4 (Bates *et al.*, 2015) and MASS packages (Ripley *et al.*, 2023). The *P*-values were obtained via the Wald χ^2^ test using the ‘ANOVA’ function from the car package (Fox and Weisberg, 2019), and a value of <0.05 was used to infer statistical significance. The ‘glht’ function from the multcomp package was used for Tukey’s post-hoc test (Hothorn *et al.*, 2008).

## RESULTS

### Temperature conditions

**Fig. 3.**
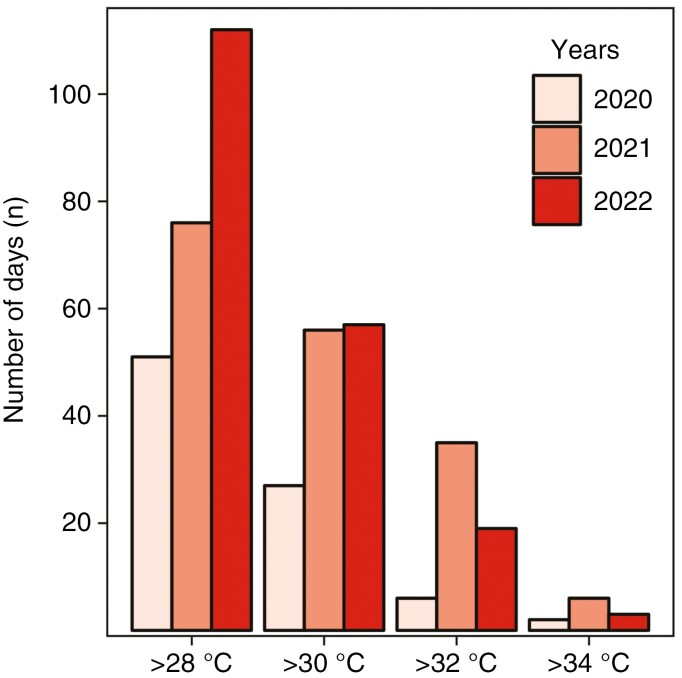
A barplot representing the number of days for which the temperature exceeded specific thresholds (28, 30, 32 and 34 °C) in 2020, 2021 and 2022.

**Fig. 4.**
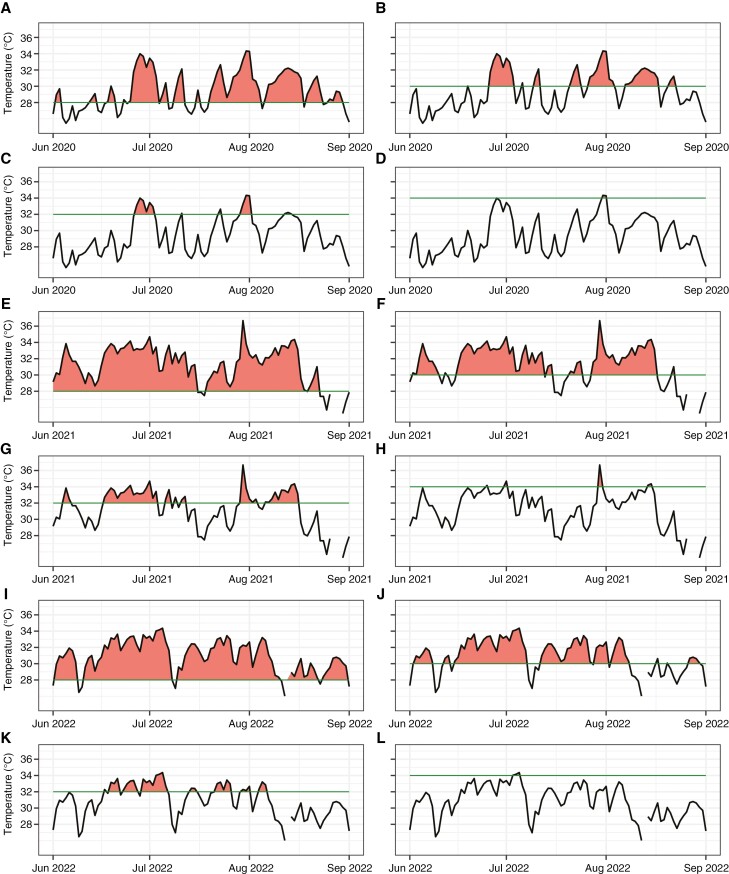
Exceedance of the 28, 30, 32 and 34 °C thresholds in the period from 1 June to 1 September for 2020 (A–D), 2021 (E–H) and 2022 (I–L).

In 2020, the temperature exceeded 28 °C for a total of 51 days, 30 °C for 27 days, 32 °C for 6 days and 34 °C for 2 days (Fig. 3). The longest period of temperature exceeding 28 °C occurred for 42 days consecutively (20 June–30 August; Fig. 4A). Likewise, the longest period of temperature exceeding 30 °C lasted for 11 days consecutively (7–17 August; Fig. 4B). The temperature exceeded 32 °C for a maximum of 6 days (27 June–2 July; Fig. 4C). Finally, the longest period with temperatures exceeding 34 °C was observed for 2 days (31 July–1 August; Fig. 4D).

In 2021, the temperature exceeded 28 °C for a total of 76 days, 30 °C for 56 days, 32 °C for 35 days and 34 °C for 6 days (Fig. 3). The most prolonged period of temperature surpassing 28 °C persisted for 45 days consecutively (1 June–15 July; Fig. 4E). Furthermore, the temperature remained above 30 °C for 27 days consecutively (16 June–12 July; Fig. 4F). Additionally, the temperature exceeded 32 °C for 18 days (30 July–16 August; Fig. 4G). The exceedance of 34 °C was observed for 1 day in June, July and August, respectively (Fig. 4H).

In 2022, the temperature exceeded 28 °C for a total of 112 days, 30 °C for 57 days, 32 °C for 19 days and 34 °C for 3 days (Fig. 3). The longest period of temperature surpassing 28 °C spanned 70 days consecutively (2 June–10 August; Fig. 4I). Likewise, the temperature remained higher than 30 °C for 23 days consecutively (15 June–7 July; Fig. 4J). Furthermore, the temperature exceeded 32 °C for 14 days (23 June–6 July; Fig. 4K). Lastly, the exceedance of 34 °C persisted for 3 days (7 July–9 July; Fig. 4L). The data are presented in Supplementary Data (Tables S1–S4).

When comparing the temperature conditions outside the lagoon with the *in situ* temperatures from the lagoon, *in situ* data show that mean seawater temperatures are considerably higher in the warmer part of the year, whereas they are considerably lower in the colder part of the year (Supplementary Data Fig. S1). Furthermore, when considering SST data beyond the lagoon and examining the time frame from the early 1980s to the present, a conspicuous trend becomes apparent: the frequency, duration (Supplementary Data Fig. S4) and intensity (Supplementary Data Fig. S5) of MHWs have increased markedly over the decades. When comparing the frequency of MHW events of the decade 2013–2022 with frequencies of events in 1983–1992, 1993–2002 and 2003–2013, the frequency has increased by 14.6, 4 and 2.1 times, respectively. Moreover, when summing the duration of MHWs in each decade, in the last decade the duration has increased by 19.7, 4.5 and 2.7 times, respectively, and when summing the cumulative intensity (which is a function of the duration and mean intensity of MHW events), it has increased by 19.5, 4.6 and 2.5 times, respectively.

### The thermotolerance experiment

**Fig. 5.**
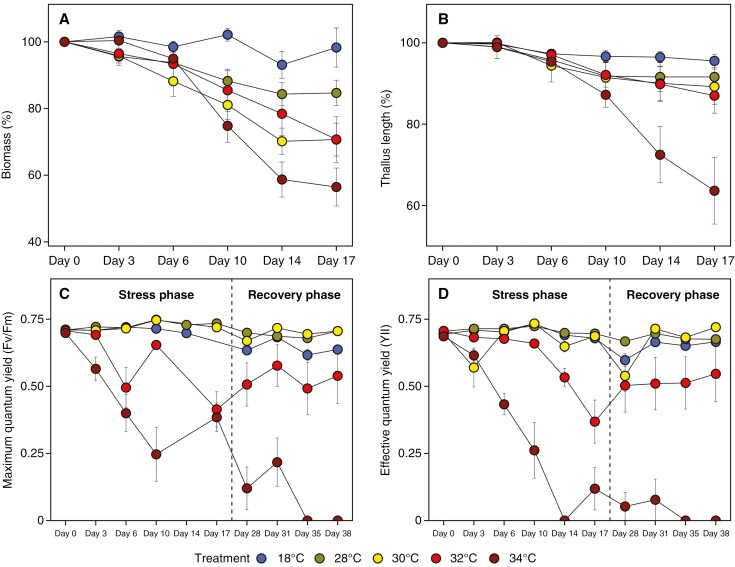
Impact of different treatments in the 2023 thermotolerance experiment on the change of: (A) biomass (mean ± s.e.); (B) thallus length (mean ± s.e.); (C) maximum quantum yield (*Fv*/*Fm*; mean ± s.e.); and (D) effective quantum yield (*YII*; mean ± s.e.). The *Fv*/*Fm* and *YII* were measured and visualized in both the stress and recovery phases (C, D). The means were calculated based on the sample size, *n* = 9.

The biomass for individuals in the 18 °C treatment remained relatively consistent throughout the entire experiment in comparison to the four treatments (Fig. 5A). Individuals in the 28 °C treatment exhibited values higher than 80 % of the initial values by the end of the experiment, whereas those in the 30 and 32 °C treatments dropped below 80 %, and individuals in the 34 °C treatment dropped below 60 % (Fig. 5A). The only significant difference in biomass was detected between the 34 and 18 °C treatments (Supplementary Data Table S5). The thallus length was relatively consistent and comparable for individuals in the 18, 28, 30 and 32 °C treatments (Fig. 5B). However, there was a drastic decrease in thallus length for individuals in the 34 °C treatment on days 14 and 17, resulting in a final value of 63.63 % (Fig. 5B). Significant differences were detected between the 34 °C treatment and both the 18 and 28 °C treatments (Supplementary Data Table S5).

The *Fv*/*Fm* (Fig. 5C) and *YII* (Fig. 5D) remained consistent throughout the entire experiment for individuals in the 18, 28 and 30 °C treatments. However, greater variability was observed in the 32 and 34 °C treatments. Individuals in the 34 °C treatment exhibited a decreasing trend in both *Fv*/*Fm* and *YII* (with very little or no recovery potential). The 32 °C treatment also showed a decrease, but without a further decline in the recovery phase, when the yield values stabilized. Significant differences were found for the *Fv*/*Fm* between the 34 and 18 °C treatments, 32 and 28 °C treatments, 34 and 28 °C treatments, 32 and 30 °C treatments and 34 and 30 °C treatments (Supplementary Data Table S5). Additionally, significant differences in *YII* were detected between the 32 and 18 °C treatments, 32 and 28 °C treatments, and between the 34 °C treatment and all other treatments (Supplementary Data Table S5).

### Visual observations

**Fig. 6.**
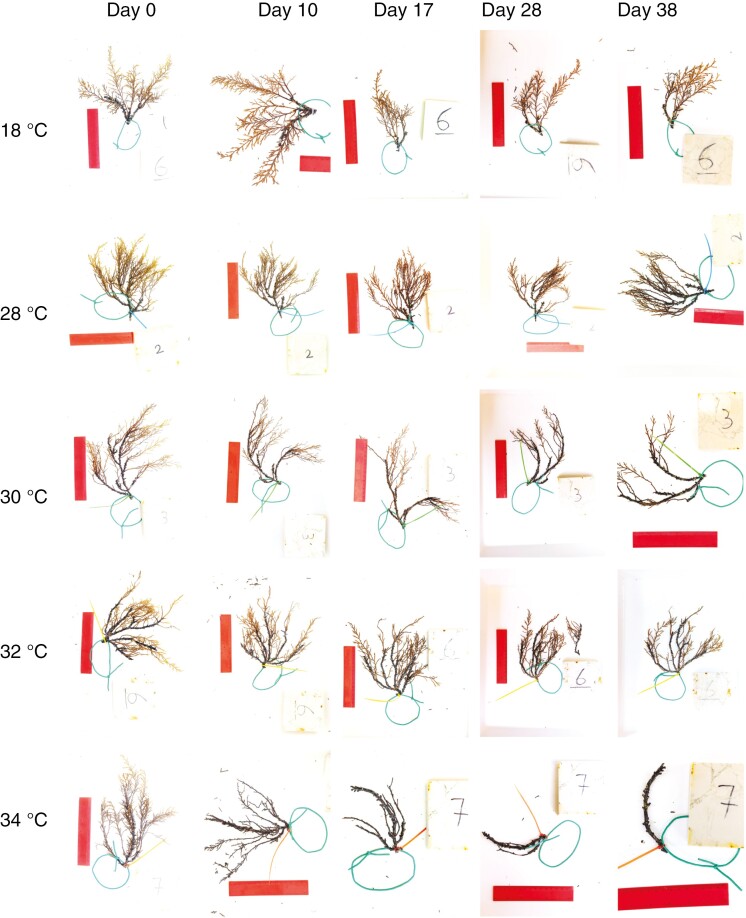
Photographs of selected individuals from the thermotolerance experiment, exposed to different temperature treatments (28, 30, 32 and 34 °C) and changes in appearance at different measurement intervals.

On day 10, a very slight necrosis on the tips of branches was noticeable at 28–32 °C, but substantial thallus damage was observed on algae at 34 °C (Fig. 6). On day 17, no noticeable changes were observed at 28 and 30 °C, but more branch necrosis and decomposition were observed at 32 °C. The algae exposed to 34 °C had lost almost all branches, while cauloid colour started to darken noticeably. Slightly more pronounced necrosis and decomposition was noticed on day 28 in the algae exposed to 28–32 °C, but no major thallus damage was observed. Algae exposed to 34 °C degraded further, with observed cauloid disintegration. Slight branch necrosis was observed in the 18 °C treatment. On day 38, the same patterns were observed, but some of the algae exposed to 34 °C had died. Interestingly, evidence of recruitment was also observed underneath the algae that contained fertile receptacles at all temperatures, but only recruits found in the 18 °C group tanks survived until the end of the experiment.

## DISCUSSION

In this experiment, we observed that *G. barbata* demonstrates a remarkable resilience to climate extremes, withstanding temperatures as high as 32 °C and demonstrating a remarkable capacity for both morphological and physiological recovery following prolonged exposure to heat stress. Individuals tested here show a thermal tolerance higher than that previously recorded for the species, with thresholds having been estimated at 23 °C (Ercegović, 1952) and ≤30 °C in isolated cases (Orfandis, 1991; Iveša *et al.*, 2022). Furthermore, exploring the MHWs on the southern Istrian coast, including the period from 1983 to 2023, our results showed that the frequency, duration and intensity of marine heatwaves has increased markedly in the last decade in comparison to prior ones. Proper detection of MHWs, following the definition by Hobday *et al.* (2016, 2018), inside Šćuza Lagoon is not possible currently because of the lack of long-term temperature data, and SST might be inappropriate for capturing small-scale variability or extreme temperature events in some settings (Smale and Wernberg, 2009; Stobart *et al.*, 2016). However, by analysing trends observed beyond the lagoon through satellite-derived data, we can infer that the conditions in the lagoon are likely to be following a similar trajectory. In line with prevailing global trends, forecasting the escalating frequency and intensity of MHWs in the forthcoming decades (Smith *et al.*, 2023), the evaluation of temperature thresholds for the population of *G. barbata* in the Šćuza coastal lagoon, recently designated as a refuge habitat, is of crucial conservation concern, notably considering its status as one of the last surviving forests along the Istrian coast.

During sampling, the temperature in the lagoon was ~12–13 °C, with *G. barbata* exhibiting well-developed branches and receptacles at this time. However, during our experiment we increased the temperature gradually to simulate spring/autumn (18 °C) and summer (28, 30, 32 and 34 °C) conditions. Extremely high temperatures and extremely low temperatures are observed in the lagoon on a yearly basis. Except for the pronounced seasonal variations, extreme temperature fluctuations were also observed daily as a result of diurnal variation in atmospheric and seawater conditions, which is typical for shallow environments. These daily temperature ranges sometimes exceeded 13 °C (Supplementary Data Fig. S2) in the summer period, and even more in colder seasons. Our MHW scenario of 17 days is realistic in the context of recorded maximum values, but we have to take into consideration that daily temperature fluctuations could play a pivotal role in mitigating and compensating for the impact of extreme temperatures in the field (Csordas *et al.*, 2023). Responses to heating events can vary not only as a consequence of MHW characteristics but also among populations and individuals based on disturbance history and local adaptations (Smith *et al.*, 2023). Nevertheless, results from our fixed temperature approach suggest that individuals from the lagoon are probably adapted to even more extreme scenarios than currently experienced in the field and will probably withstand further warming. It is, however, important to note that some authors consider including only fixed thermal thresholds as over-simplistic and suggest simulations in which sustained warming and MHWs with different levels of variable temperature profiles should be considered, because the highest temperature treatment does not always trigger the strongest effect (Straub *et al.*, 2022).

Temperature stress has the capacity to disrupt cellular biological processes (Davison and Pearson, 1996) and impact performance (Crafts-Brandner and Salvucci, 2002), including the functionality of photosynthetic apparatus (Bischof and Rautenberger, 2012). In our study, the potential for recuperation of photosynthetic performance was noted in individuals exposed to temperatures as high as 32 °C. We hypothesize that clear water and high light availability could have partly offset an expected negative impact of temperature stress on photosynthetic yield. A recent study has shown that some cold-water kelps in high-light conditions were resistant to simulated MHW activity (Bass *et al.*, 2023). In the study area here, we observed that sedimentation rates are high (potentially limiting the light availability); however, given the shallow positioning of this population within the lagoon, exposure to higher light intensities might mitigate any expected adverse effects.

*Gongolaria barbata* shows an annual growth cycle in which perennial (cauloid) material typically exhibits continuous growth, with higher growth rates in colder seasons and slower rates in warmer seasons. However, according to Ercegović (1952), in sheltered areas such as bays and lagoons, where water circulation is limited, *G. barbata* experiences an aestivation period during summer owing to intense warming conditions (Iveša *et al.*, 2022). This dormancy can persist for an extended period of ≤3 months, during which time annual branch material is also lost. We have observed this pattern both *in situ* and *ex situ* and hypothesize that regeneration is triggered by lowering of the temperature conditions. At the conclusion of this experiment (after 38 days), all individuals were transferred to stone basins and exposed to constant natural seawater flow, which at the time was ~15 °C. We observed signs of regeneration and regrowth of branches from cauloids in a span of a few weeks, indicating the ability of individuals to tolerate, survive and regenerate morphologically even after prolonged exposure to extreme temperatures. Notably, however, regrowth was limited amongst individuals previously exposed to the 34 °C temperature treatment, in which only two of nine individuals showed signs of regrowth, suggesting a possible temperature threshold for recovery.

Although summer extremes and prolonged exposure above temperature thresholds are expected to lead to increased annual mortality of adult individuals in the lagoon, MHWs could additionally disrupt the seasonal vegetative cycle of the species, resulting in various other responses (Bevilacqua *et al.*, 2019). Temperature influences the reproductive potential and recruitment processes for a range of seaweed species (Bartsch *et al.*, 2013; Andrews *et al.*, 2014; de Bettignies *et al.*, 2018; Muth *et al.*, 2019). For example, ocean warming has been linked to decreased reproductive output and higher than expected sporophyte mortality (Breeman, 1988; Ladah and Zertuche-González, 2007; Wernberg *et al.*, 2011). Locally, an MHW that occurred in February 2019 in the northern Adriatic Sea caused a phenological shift that triggered early fertility of *G. barbata* in some settlements (Bevilacqua *et al.*, 2019), and we do not yet understand the consequences of such a shift for long-term population dynamics (Csordas *et al.*, 2023). In our study, at the time of collection, nearly all individuals were fertile, and we observed that during the acclimation period, gamete release was induced at ~18 °C and lead to successful fertilization and settlement of zygotes. Interestingly, however, recruits survived only in control conditions (held at a constant 18 °C). Detrimental effects of elevated temperatures (28 °C) on recruits have also been observed in *Ericaria crinita* (Verdura *et al.*, 2021), a Mediterranean canopy-forming species sharing some similar life-history traits with *G. barbata*. Conversely however, in other species, early life-history adaptability has been shown under temperature stress, for example warm-acclimated *Saccharina latissima* sporophytes exhibited enhanced photosynthetic efficiency and required less light to achieve maximum photosynthetic rates at high temperatures (Davison *et al.*, 1991), and warm-acclimated *Fucus vesiculosus* embryos showed increased survival rates during periods of thermal stress (Li and Brawley, 2004). Given the uniqueness of the studied population in Šćuza Lagoon, similar studies should be performed here and for other refuge-type habitats (shallow bays and rockpools), aiming to relate heat resilience to genetic features and phylogeography (Liesner *et al.*, 2020).

Currently, there is a particular interest in understanding the role of temperature stress on the early life stages of *Cystoseira s.l.* species because the loss of forest cover is now widespread throughout the Mediterranean (Thibaut *et al.*, 2015) and active restoration is gaining momentum as a conservation measure (Cebrian *et al.*, 2021). It is unfortunate that as a seed source, the *G. barbata* population in Šćuza Lagoon is isolated geographically and not well connected to the wider Adriatic (Iveša *et al.*, 2022). *Gongolaria barbata* is known to have low dispersal capacity (Verdura *et al.*, 2018), and thus the species distribution is unlikely to expand beyond the lagoon unless facilitated via active restoration. Given the extreme temperatures measured in Šćuza Lagoon and the unexpected survival of *G. barbata* in this environment, this population is a potentially thermally tolerant source of restoration material, potentially improving the likelihood for longevity of any restoration attempts. However, further investigation is required to determine whether this population is a unique ecotype, having evolved its observed thermal adaptation over many generations or more rapidly through exposure to acute thermal stress (King *et al.*, 2018; Coleman and Wernberg, 2020). Crucially, the benefit of research of this nature is 2-fold: firstly, we can optimize outplant timing to improve the chances of restoration success (Cebrian *et al.*, 2021); and secondly, we can understand the vulnerability of this rare refuge population. Furthermore, the state, role and interaction of factors such as oxygen, light, nutrient levels, eutrophication and extreme low tides remain unknown. Additionally, the role of epibionts (we observe a high cover of epibionts on *G. barbata* individuals during the summer) and herbivores (e.g. known contributors to the decline of canopy-forming species; Iveša *et al.*, 2016, 2022) is not well understood and will require further investigation.

## Conclusion

According to our results, the *G. barbata* population inhabiting the Šćuza coastal lagoon is resilient and should be highly adaptable in response to the predicted increase in frequency and intensity of extreme environmental conditions. Therefore, given the decline of *G. barbata* and related species along the coast, the Šćuza population can potentially serve as a donor site, providing valuable thermally tolerant reproductive material useful for local restoration and conservation efforts in the Adriatic Sea. Furthermore, the Šćuza Lagoon provides a valuable natural laboratory for investigating resilience and evolving life histories of *G. barbata* and related species to extreme temperatures. Such research is crucial given the rapidly progressing field of restoration science, including for Mediterranean *Cystoseira s.l.* species (Orlando-Bonaca *et al.*, 2021; Savonitto *et al.*, 2021; Galobart *et al.*, 2023; Kaleb *et al.*, 2023).

## SUPPLEMENTARY DATA

Supplementary data are available at *Annals of Botany* online and consist of the following.

Figure S1: *In situ* daily mean temperatures in the lagoon (red line) and satellite (SST) daily mean temperatures outside the lagoon (blue line), in the period from 2020 to the end of 2022. Figure S2: Daily temperature variations in the period when the highest temperatures were detected in the lagoon (2022). Maxima are presented in red, minima in blue, and mean daily temperatures in black. Figure S3: Scheme of the thermotolerance experimental set-up. Figure S4: Duration and frequency of MHWs in the period from 1983 to 2023 [satellite-derived sea surface temperature data (OISST v.2) from the ERDDAP server], on the southern Istrian coast. Figure S5: Intensity and frequency of heatwaves in the period from 1983 to 2023 [satellite-derived sea surface temperature data (OISST v.2) from the ERDDAP server], on the southern Istrian coast. Table S1: duration (in days) of periods when the temperature remained above the 28 °C threshold in Šćuza for 2020, 2021 and 2022. Table S2: duration (in days) of periods when the temperature remained above the 30 °C threshold in Šćuza for 2020, 2021 and 2022. Table S3: duration (in days) of periods when the temperature remained above the 32 °C threshold in Šćuza for 2020, 2021 and 2022. Table S4: duration (in days) of periods when the temperature remained above the 34 °C threshold in Šćuza for 2020, 2021 and 2022. Table S5: analyses of deviance (Wald χ^2^ test) for each fitted model used to test the impact of temperature on morphological and physiological characteristics of *Gongolaria barbata*, and summary statistics from Tukey’s post-hoc test for the pairwise comparisons between different levels of the fixed factor. *P < 0.05, **P < 0.01 and ***P < 0.001.

mcae038_suppl_Supplementary_Material
