# Correction to: Effects of extreme temperatures and recovery potential of *Gongolaria barbata* from a coastal lagoon in the northern Adriatic Sea: an *ex situ* approach

**DOI:** 10.1093/aob/mcae070

**Published:** 2024-05-08

This is a correction to: Andrea Bilajac, Edi Gljušćić, Shannen Smith, Mirjana Najdek, Ljiljana Iveša, Effects of extreme temperatures and recovery potential of *Gongolaria barbata* from a coastal lagoon in the northern Adriatic Sea: an *ex situ* approach, Annals of Botany, 2024;, mcae038, https://doi.org/10.1093/aob/mcae038

Upon original publication, the order of Supplementary Data Figures S1-S5 in the supplementary file accompanying this article was incorrect.

The supplementary file of the published article has been updated with the correct version.

