# Supplementary material for: Effects of extreme temperatures and recovery potential of Gongolaria barbata from a coastal lagoon in the northern Adriatic Sea: an ex situ approach
Source: Ann Bot. 2024 Mar 14;134(3):415–26. doi: 10.1093/aob/mcae038 (PMC11341668; doi:10.1093/aob/mcae038)
Supplement: mcae038_suppl_Supplementary_Material [file mcae038_suppl_supplementary_material.docx]

**Supplementary Data - Bilajac *et al.* 2024**

**Figure S1.** *In situ* daily mean temperatures in Šćuza Lagoon (red line) and satellite (SST) daily mean temperatures outside the lagoon (blue line), in the period from 2020 to the end of 2022.


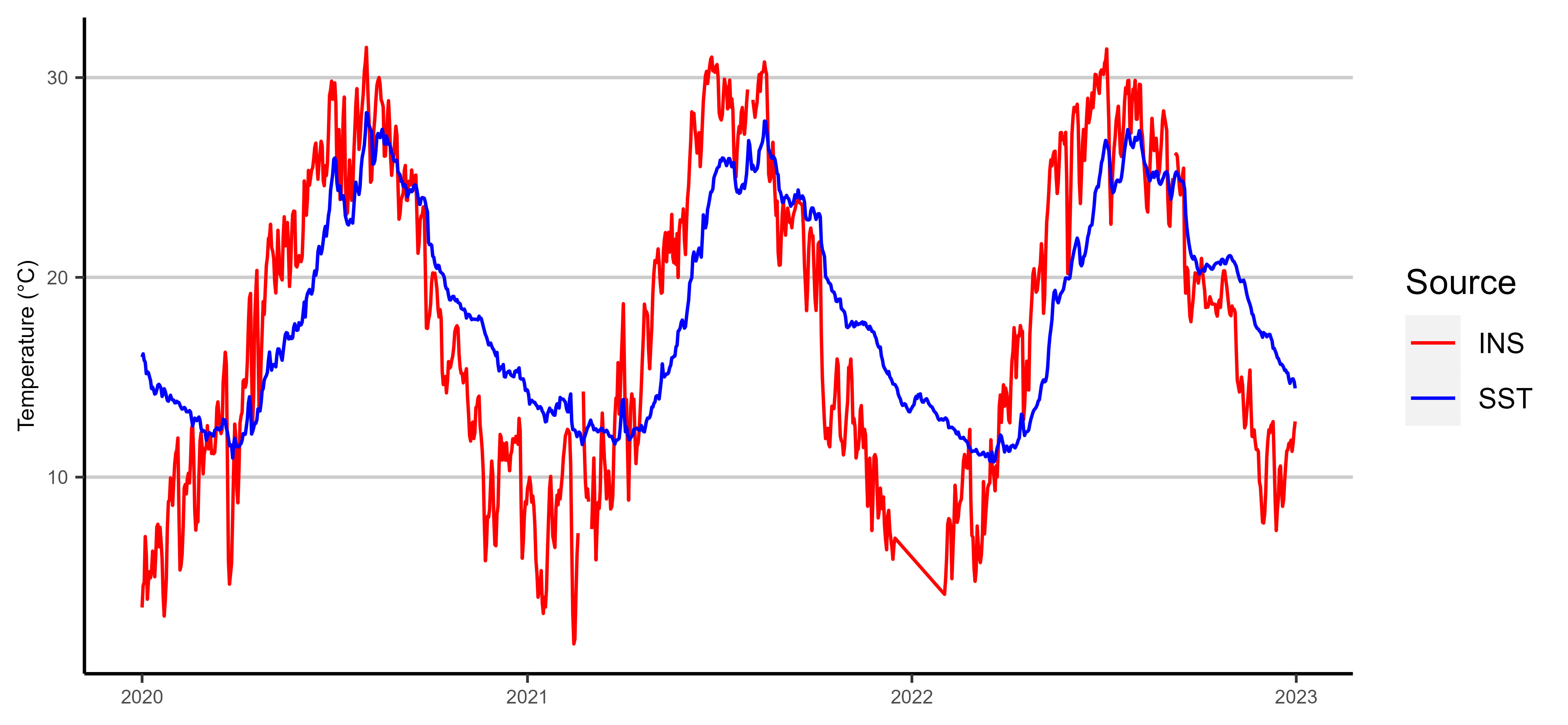


**Figure S2.** Daily temperature variations in the period when the highest temperatures were detected in the lagoon (2 June 2022 – 10 Aug 2022). Maxima are presented in red, minima in blue, while mean daily temperatures in black.


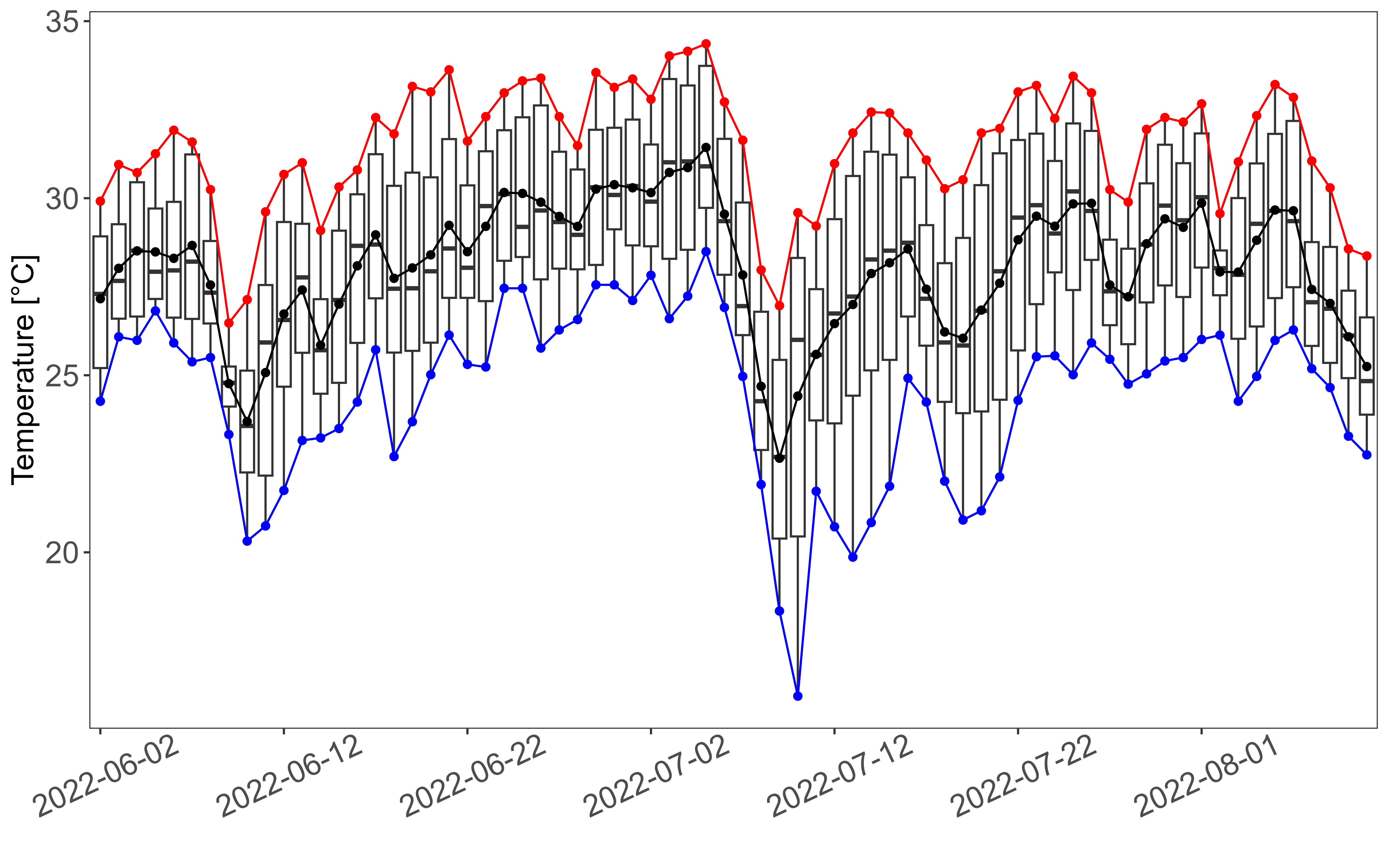


**Figure S3.** Scheme of the thermotolerance experimental setup.


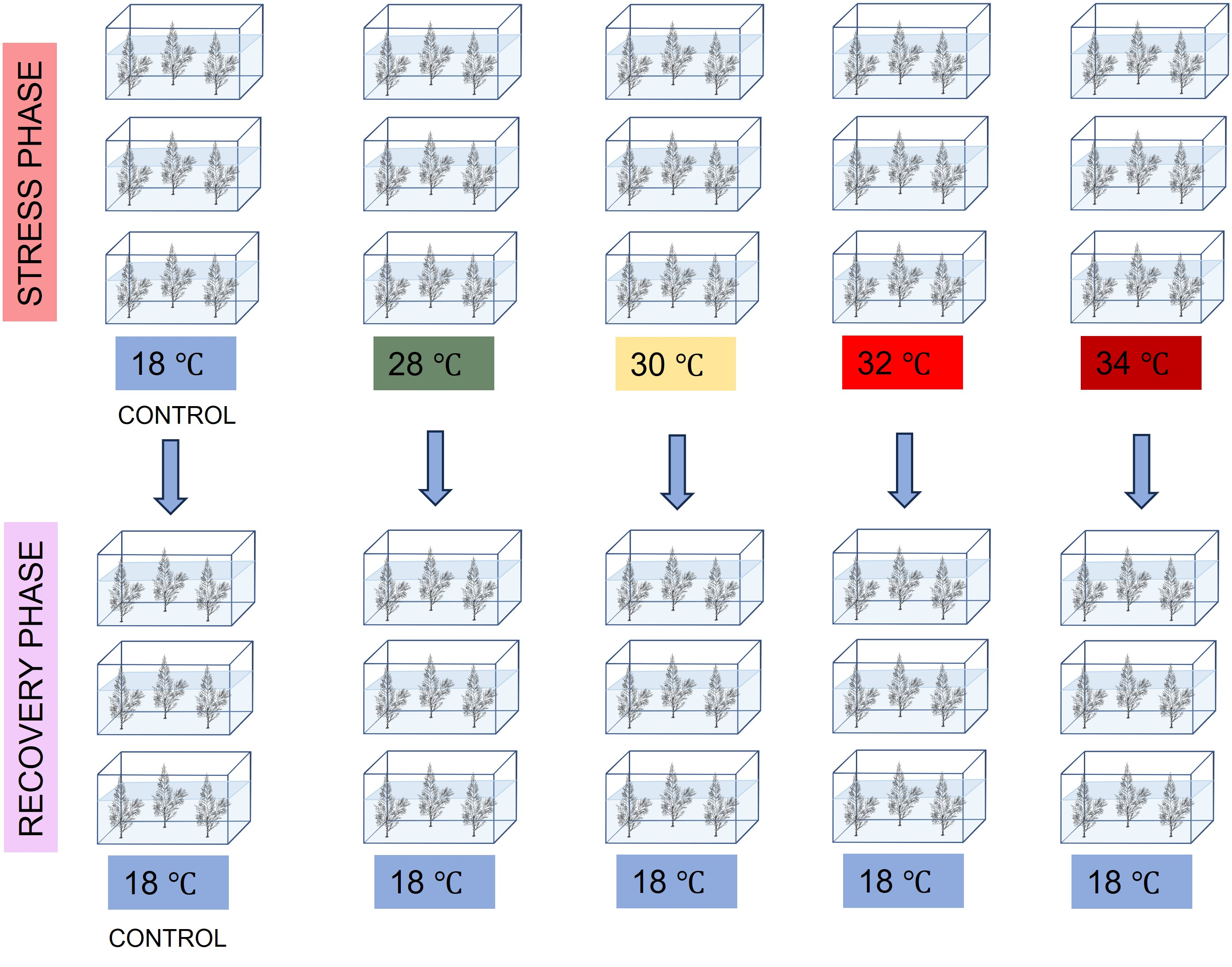


**Figure S4.** Duration and frequency of MHWs in the period from 1983 to 2023 (Satellite-derived Sea Surface Temperature data (OISST v.2) from the ERDDAP server), on the southern Istrian coast.


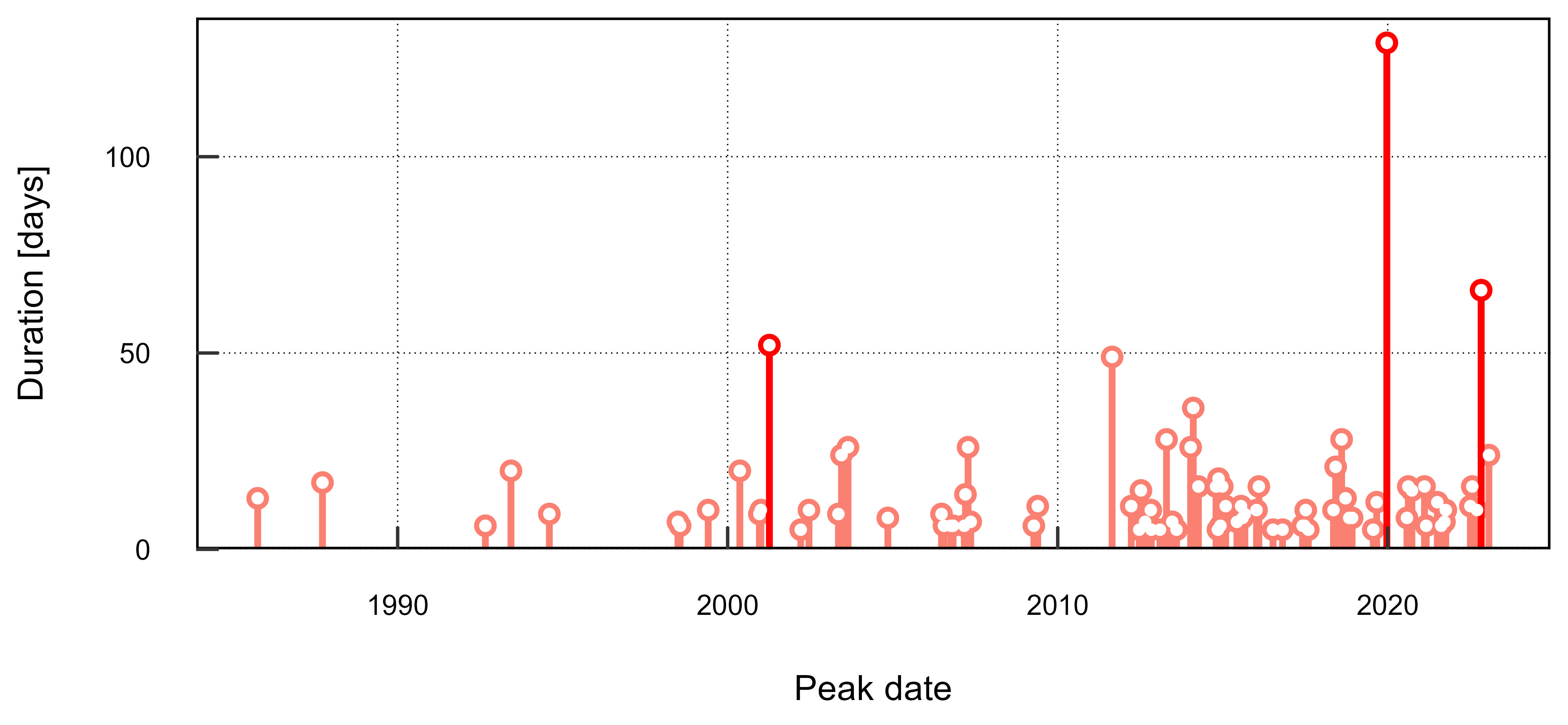


**Figure S5.** Intensity and frequency of MHWs in the period from 1983 to 2023 (Satellite-derived Sea Surface Temperature data (OISST v.2) from the ERDDAP server), on the southern Istrian coast.


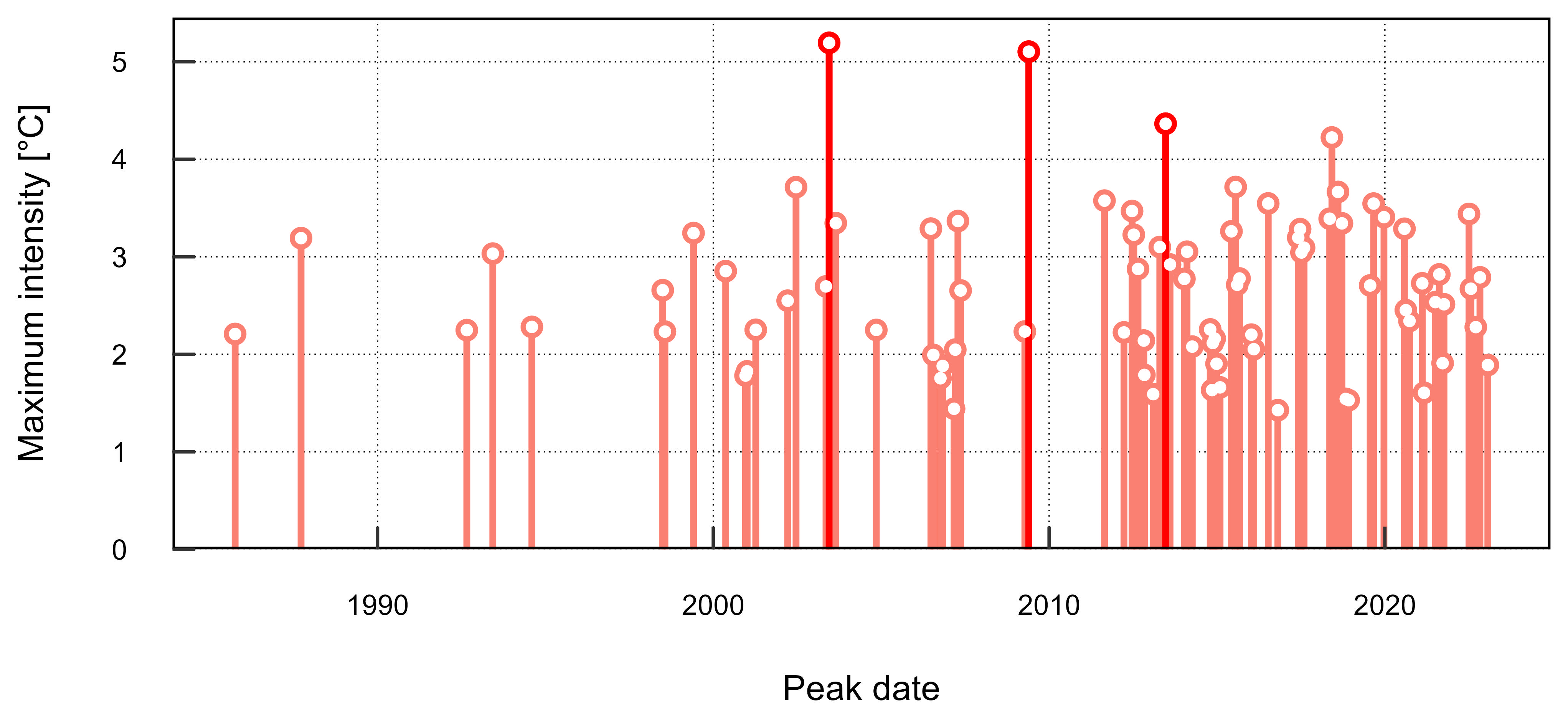


**Table S1.** Duration (days) of periods when the temperature remained above the 28 °C threshold in Šćuza for years 2020, 2021 and 2022.

| **Duration** | **Date_start** | **Date_peak** | **Date_end** | **Temp** | **Year** |
| --- | --- | --- | --- | --- | --- |
| 9 | 2020-06-25 | 2020-06-28 | 2020-07-03 | 28 | 2020 |
| 42 | 2020-07-20 | 2020-07-31 | 2020-08-30 | 28 | 2020 |
| 45 | 2021-06-01 | 2021-07-01 | 2021-07-15 | 28 | 2021 |
| 31 | 2021-07-19 | 2021-07-30 | 2021-08-18 | 28 | 2021 |
| 19 | 2022-05-09 | 2022-05-22 | 2022-05-27 | 28 | 2022 |
| 70 | 2022-06-02 | 2022-07-05 | 2022-08-10 | 28 | 2022 |
| 18 | 2022-08-14 | 2022-08-28 | 2022-08-31 | 28 | 2022 |
| 5 | 2022-09-06 | 2022-09-07 | 2022-09-10 | 28 | 2022 |

**Table S2.** Duration (days) of periods when the temperature remained above the 30 °C threshold in Šćuza for years 2020, 2021 and 2022.

| **Duration** | **Date_start** | **Date_peak** | **Date_end** | **Temp** | **Year** |
| --- | --- | --- | --- | --- | --- |
| 8 | 2020-06-26 | 2020-06-28 | 2020-07-03 | 30 | 2020 |
| 8 | 2020-07-27 | 2020-07-31 | 2020-08-03 | 30 | 2020 |
| 11 | 2020-08-07 | 2020-08-13 | 2020-08-17 | 30 | 2020 |
| 9 | 2021-06-02 | 2021-06-05 | 2021-06-10 | 30 | 2021 |
| 27 | 2021-06-16 | 2021-07-01 | 2021-07-12 | 30 | 2021 |
| 20 | 2021-07-28 | 2021-07-30 | 2021-08-16 | 30 | 2021 |
| 6 | 2022-05-20 | 2022-05-22 | 2022-05-25 | 30 | 2022 |
| 6 | 2022-06-03 | 2022-06-06 | 2022-06-08 | 30 | 2022 |
| 23 | 2022-06-15 | 2022-07-05 | 2022-07-07 | 30 | 2022 |
| 16 | 2022-07-12 | 2022-07-25 | 2022-07-27 | 30 | 2022 |
| 6 | 2022-08-03 | 2022-08-05 | 2022-08-08 | 30 | 2022 |

**Table S3.** Duration (days) of periods when the temperature remained above the 32 °C threshold in Šćuza for years 2020, 2021 and 2022.

| **Duration** | **Date_start** | **Date_peak** | **Date_end** | **Temp** | **Year** |
| --- | --- | --- | --- | --- | --- |
| 6 | 2020-06-27 | 2020-06-28 | 2020-07-02 | 32 | 2020 |
| 17 | 2021-06-17 | 2021-07-01 | 2021-07-03 | 32 | 2021 |
| 18 | 2021-07-30 | 2021-07-30 | 2021-08-16 | 32 | 2021 |
| 14 | 2022-06-23 | 2022-07-05 | 2022-07-06 | 32 | 2022 |
| 5 | 2022-07-22 | 2022-07-25 | 2022-07-26 | 32 | 2022 |

**Table S4.** Duration (days) of periods when the temperature remained above the 34 °C threshold in Šćuza for years 2020, 2021 and 2022.

| **Duration** | **Date_start** | **Date_peak** | **Date_end** | **Temp** | **Year** |
| --- | --- | --- | --- | --- | --- |
| 2 | 2020-07-31 | 2020-07-31 | 2020-08-01 | 34 | 2020 |
| 1 | 2021-02-21 | 2021-02-21 | 2021-02-21 | 34 | 2021 |
| 1 | 2021-06-25 | 2021-06-25 | 2021-06-25 | 34 | 2021 |
| 1 | 2021-07-01 | 2021-07-01 | 2021-07-01 | 34 | 2021 |
| 1 | 2021-07-30 | 2021-07-30 | 2021-07-30 | 34 | 2021 |
| 2 | 2021-08-14 | 2021-08-15 | 2021-08-15 | 34 | 2021 |
| 3 | 2022-07-03 | 2022-07-05 | 2022-07-05 | 34 | 2022 |

**Table** **S5.** Analyses of deviance (Wald ꭓ2 test) for each fitted model used to test the impact of temperature on morphological and physiological characteristics of *Gongolaria barbata*, and summary statistics from Tukey test for the pairwise comparisons between different levels of the fixed factor. „*“ denotes the 0.05 significance level, „**“ denotes the the 0.01 significance level and „***“ denotes the 0.001 significance level.

| Effect of fixed factor/temperature | | | | | Tukey post-hoc test | | |
| --- | --- | --- | --- | --- | --- | --- | --- |
| Test | Response | ꭓ2 | Df | p | Pairwise | z-value | p |
| LMM | Biomass | 11.275 | 4 | 0.023 * | 28C - 18C | -1.342 | 0.665 |
|  |  |  |  |  | 30C - 18C | -2.503 | 0.090 |
|  |  |  |  |  | 32C - 18C | -1.972 | 0.280 |
|  |  |  |  |  | 34C - 18C | -3.090 | 0.017 * |
|  |  |  |  |  | 30C - 28C | -1.161 | 0.773 |
|  |  |  |  |  | 32C - 28C | -0.630 | 0.970 |
|  |  |  |  |  | 34C - 28C | -1.749 | 0.404 |
|  |  |  |  |  | 32C - 30C | 0.531 | 0.984 |
|  |  |  |  |  | 34C - 30C | -0.587 | 0.977 |
|  |  |  |  |  | 34C - 32C | -1.118 | 0.797 |
| GLMM | Length | 13.993 | 4 | 0.003 ** | 28C - 18C | -0.860 | 0.911 |
|  |  |  |  |  | 30C - 18C | -1.112 | 0.801 |
|  |  |  |  |  | 32C - 18C | -1.068 | 0.823 |
|  |  |  |  |  | 34C - 18C | -3.783 | 0.001 ** |
|  |  |  |  |  | 30C - 28C | -0.252 | 0.999 |
|  |  |  |  |  | 32C - 28C | -0.208 | 1.000 |
|  |  |  |  |  | 34C - 28C | -2.924 | 0.028 * |
|  |  |  |  |  | 32C - 30C | 0.044 | 1.000 |
|  |  |  |  |  | 34C - 30C | -2.673 | 0.058 |
|  |  |  |  |  | 34C - 32C | -2.717 | 0.052 |
| GLMM | *Fv/Fm* | 89.595 | 4 | < 2.2e-16 *** | 28C - 18C | 0.883 | 0.903 |
|  |  |  |  |  | 30C - 18C | 0.777 | 0.937 |
|  |  |  |  |  | 32C - 18C | -2.629 | 0.065 |
|  |  |  |  |  | 34C - 18C | -7.040 | < 0.001 *** |
|  |  |  |  |  | 30C - 28C | -0.107 | 1.000 |
|  |  |  |  |  | 32C - 28C | -3.512 | 0.004 ** |
|  |  |  |  |  | 34C - 28C | -7.919 | < 0.001 *** |
|  |  |  |  |  | 32C - 30C | -3.406 | 0.006 ** |
|  |  |  |  |  | 34C - 30C | -7.813 | < 0.001 *** |
|  |  |  |  |  | 34C - 32C | -4.418 | < 0.001 *** |
| GLMM | *Y(II)* | 117.12 | 4 | < 2.2e-16 *** | 28C - 18C | 1.276 | 0.706 |
|  |  |  |  |  | 30C - 18C | -0.635 | 0.969 |
|  |  |  |  |  | 32C - 18C | -2.894 | 0.031 * |
|  |  |  |  |  | 34C - 18C | -8.602 | <0.001 *** |
|  |  |  |  |  | 30C - 28C | -1.911 | 0.311 |
|  |  |  |  |  | 32C - 28C | -4.165 | <0.001 *** |
|  |  |  |  |  | 34C - 28C | -9.825 | <0.001 *** |
|  |  |  |  |  | 32C - 30C | -2.262 | 0.157 |
|  |  |  |  |  | 34C - 30C | -7.994 | <0.001 *** |
|  |  |  |  |  | 34C - 32C | -5.803 | <0.001 *** |
